# Poor quality in the reporting and use of statistical methods in public health – the case of unemployment and health

**DOI:** 10.1186/s13690-015-0096-6

**Published:** 2015-11-16

**Authors:** Fredrik Norström

**Affiliations:** Department of Public Health and Clinical Medicine, Epidemiology and Global Health, Umeå University, SE-901 87 Umeå, Sweden

**Keywords:** Review

## Abstract

**Background:**

It has previously been reported that many research articles fail to fulfill important criteria for statistical analyses, but, to date, these reports have not focused on public health problems. The aim of this study was to investigate the quality of reporting and use of statistical methods in articles analyzing the effect of unemployment on health.

**Methods:**

Forty-one articles were identified and evaluated in terms of how they addressed 12 specified criteria.

**Results:**

For most of these criteria, the majority of articles were inadequate. These criteria were conformity with a linear gradient (100 % of the articles), validation of the statistical model (100 %), collinearity of independent variables (97 %), fitting procedure (93 %), goodness of fit test (78 %), selection of variables (68 % for the candidate model; 88 % for the final model), and interactions between independent variables (66 %). Fewer, but still alarmingly many articles, failed to fulfill the criteria coefficients presented in statistical models (48 %), coding of variables (34 %) and discussion of methodological concerns (24 %). There was a lack of explicit reporting of statistical significance/confidence intervals; 34 % of the articles only presented *p*-values as being above or below the significance level, and 42 % did not present confidence intervals. Events per variable was the only criterion met at an undoubtedly acceptable level (2.5 %).

**Conclusions:**

There were critical methodological shortcomings in the reviewed studies. It is difficult to obtain unbiased estimates, but there clearly needs to be some improvement in the quality of documentation on the use and performance of statistical methods. A suggestion here is that journals not only demand that articles fulfill the criteria within the STROBE statement, but that they include additional criteria to decrease the risk of incorrect conclusions being drawn.

**Electronic supplementary material:**

The online version of this article (doi:10.1186/s13690-015-0096-6) contains supplementary material, which is available to authorized users.

## Background

How well statistical methods are performed is essential to obtain reliable results. Failure in data analysis could lead to incorrect conclusions being drawn having serious negative implications, for example, in the treatment of patients. Poor quality in the reporting and performance of statistical analyses has been reported numerous times in scientific papers [1–23]. Problems have been reported in regard to multivariable methods in general [21], logistic regression [2, 5, 7, 9, 11, 16, 17, 19], the chi-square test [12], and for the treatment of confounders in the statistical model [6, 18]. The poor quality is not restricted to journals with low impact factors, as it is also common in journals with high impact factors [18]. Methodological problems were reported over 30 years ago [23], and despite continued efforts to improve the situation [24], improvements have been far from satisfactory [9, 15, 25]. Poor reporting and/or performance of the statistical analysis does not necessarily mean that the conclusions will be wrong. However, a key issue is that if the methods are poorly reported, readers will not be able to critically assess whether the statistical analyses provide reliable results or whether the conclusions drawn by the authors are valid. Yet these problems can be difficult to identify and there is a need for clear guidelines to inform the assessment process.

Scientific journals have instructions for authors with varied demands to oblige to before submission. For many of the journals, including Lancet and Archives of Public Health, the STROBE (“Strengthening the Reporting of Observational Studies in Epidemiology”) statement are used as the requirement for both issues related to the study design and the statistical analyses [24]. The STROBE initiative was developed to improve the quality of the reporting in observational studies, and journals requiring the STROBE statement are likely to be among the ones with highest demand related to the study design and the statistical analyses. However, the STROBE checklist has few detailed recommendations for reporting how statistical analyses were performed. The checklist is therefore not sufficient for ensuring that most of the issues related to poor quality of statistical analyses are handled correctly.

The effect of unemployment on health has been studied in numerous original papers [26–66], review articles [67–69], and meta-analyses [70–72]. It is obvious from the literature that unemployment is not good for an individual’s health, although there are contradictory results in these studies [32]. A recent review of 41 articles published from 2003 to 2014 concluded that there is limited value in an estimate of the worldwide overall effect from unemployment on health because the study context has a strong impact on the estimate of the effect size [68].

The exposure groups in a randomized controlled study have similar characteristics at the time of exposure, while this is usually not the case for observational studies. For unemployed and employed there are important differences in characteristics at the time of exposure, i.e. unemployment, and it is therefore difficult to avoid highly biased estimates for the effect of unemployment on health. The most important of these characteristics is likely to be the health status, as individuals with a poor health is more likely to get unemployed. The handling of previous health, as well as other confounding variables such as gender, age, education level, and marital status, in the statistical model not only decides if the results are reliable, but also whether results can be considered to address causality or only association. Most articles, including cross-sectional studies, are aiming to estimate the effect of unemployment on health, opt to show causality and not association. The statistical analysis method is, however, likely to limit results in many studies to at most be interpreted in terms of association. Too strong conclusions regarding causality are therefore expected to often be the case from especially cross-sectional studies. However this study does not deal with this issue of causation/association. It was not feasible to do this here because many articles lacked information essential for such an analysis.

There have been no previous evaluations of the quality of the reporting and use of statistical methods in studies which examined the relationship between health and employment status, nor are there any studies that deal with these issues within the broader field of public health. However this has been evaluated in articles in which self-rated health is the outcome variable [14]. The aim of this study was to specifically investigate the quality of reporting and the use of statistical methods in published articles analyzing the effect of unemployment on health.

## Methods

This review used the same selection criteria for articles as done in a previous review with a different aim [68]. The selection criteria for articles in the review were i) analyses from original data that aimed to measure the effect on (self-assessed) health from unemployment, ii) written in English, iii) the inclusion of a group of unemployed compared with a group of employed, iv) unemployed defined as actively searching for a job and not disabled or retired, and v) published between 2003 and April 2014. A search for articles based on these criteria was performed in the literature databases Web of Science (Thomson Reuters) and PubMed (National Center for Biotechnology Information, Bethesda, MD, USA). The procedure for the selection of the 41 articles [26–66] in this review is explained in detail in the previous review (published at http://www.biomedcentral.com/1471-2458/14/1310/additional) [68].

Many criteria have been used in the literature for assessing the quality of statistical analyses in previous articles, but this review was restricted to the criteria in Table 1. This evaluation used the criteria defined by Bagley and colleagues [2], most of which have been used several times in similar evaluations. Additionally, a criterion for the presentation of coefficients in the statistical model, previously used by Kalil and colleagues and Ottenbacher and colleagues [7, 19], and criterion 19 from the STROBE checklist requiring that methodological limitations such as potential biases are discussed [24], were included in the evaluation. For two of the criteria suggested by Bagley and colleagues, namely the presentation of statistical significance and the selection of variables, a more extensive evaluation was performed. The extensions beyond Bagley’s criteria were chosen because I observed that these important issues were poorly handled when I worked with the previous review (focusing on the effect of unemployment on health) with the same selection of articles [68].

**Table 1:** Results from the methodological evaluation of the articles in this review

Criterion	Number	Percent
*Events per variable/overfitting (n = 40)*		
Fulfilled	39	97.5 %
Not fulfilled	1	2.5 %
*Conformity with a linear gradient (n = 19)*		
Fulfilled	0	0 %
Not fulfilled	19	100 %
No continuous variable in multivariate analysis	22	
*Interactions between independent variables (n = 41)*		
Interaction terms used	14	34 %
Coefficients tested	14	
Coefficients not tested	0	
Stratified results presented	27	66 %
Test for interactions	3	
No test for interactions	24	
Neither stratified results nor interaction terms discussed	7	17 %
*Collinearity of independent variables (n = 39)*		
Discussed	1	2.6 %
Not discussed	38	97.4 %
*Validation of the statistical model (n = 41)*		
Fulfilled	0	0 %
Sensitivity analysis/robustness checks	3	7.3 %
Not used	38	92.7 %
*Statistical significance/confidence intervals (n = 41)*		
Statistical significance for statistical model	2	5.1 %
No statistical significance for statistical model	39	94.9 %
*P*-value only presented as above/below the significance level	14	34 %
Confidence interval presented for exposure	24	58 %
*Coefficients presented in statistical models with relative difference (n = 23)*		
All coefficients presented for the statistical model	13	52 %
Not all coefficients presented for the statistical model	12	48 %
*Goodness of fit test (n = 41)*		
Fulfilled	9	22 %
Not fulfilled	32	78 %
*Selection of variables (n = 41)*		
Selection of candidate variables discussed	13	32 %
Selection of candidate variables not discussed	28	68 %
Selection of final variables in statistical model discussed	5	12 %
Selection of final variables in statistical model not discussed	36	88 %
*Coding of variables (n = 41)*		
No faults	27	66 %
Small faults	11	27 %
Major faults	3	7.3 %
*Fitting procedure (n = 41)*		
Performed	3	7.3 %
Not performed	38	92.7 %
*Discussion of methodological concerns (n = 41)*		
Yes	31	76 %
Limitations with statistical model	6	15 %
No	10	24 %

### Events per variable/Overfitting

Risk estimates might be unreliable in multivariate methods if there are too few outcome events in relation to the number of independent variables [2, 5]. For logistic regression, it has been recommended based on simulation studies that the number of events for the least common of the outcomes for the binary dependent variable divided by the number of predictor variables should be at least 10 [73]. This criterion has been used as a rule of thumb for almost two decades for defining an overfitted statistical model, and it has been used in previous methodological analyses [2, 5, 7, 9, 11, 16, 17, 19] even though it might be too restrictive [74]. I used this criterion when the outcome variable was binary. For methods with a continuous outcome variable, such as linear regression, the model fit cannot be assessed in terms of events per predictor variables. I used a criterion of at least 20 observations per predictor variable for methods with a continuous or ordinal outcome variable, i.e. at least a sample size of 100 individuals if five variables were included in the statistical model. I am not aware of a recommended cut-off for these outcome variables, but even with a tougher cut-off of for example, 100 observations per predictor value, the results from this review would not have changed. The criterion was to be fulfilled for all main analyses presented in the article.

### Conformity with a linear gradient

In linear regression, an increase of one unit in a continuous predictor variable should have the same effect on the outcome variable, independent of the value of the predictor variable. For linear regression the test of normality can also be used to test if continuous variables conform to a linear gradient. For logistic regression, whether a continuous variable conforms to a linear gradient can be checked on the log-odds scale. In other regression methods, it is equally important that increases in the continuous predictor variable are in accord with the effect on the outcome variable [2, 5]. To meet this criterion, the independent variable can be grouped, but this might also create a problem because values within the group might have a different effect on the outcome variable. It is reasonable to request that the authors provide a reason for creating groups, but this was not required here. All continuous variables in the statistical model were required to fulfill the criterion of conformity to a linear gradient, and this had to be specified in the article. Articles in this review are presented as either fulfilling the criterion, not fulfilling the criterion, or having no continuous variable.

### Interactions between independent variables

Norström and colleagues showed that stratified estimates differed on a factor level [68], i.e., the predictor variables interacted. This illustrates the importance of accounting for interactions between predictor variables [2, 5]. It is important to consider all potential interactions between variables in the statistical model. Based on previous knowledge and intuitive ideas about interplaying factors, it is wise to restrict the number of interactions that are evaluated in the model. It may be prudent to not evaluate all possible interactions in a study i.e. to avoid too complex a statistical model. Nevertheless, it is reasonable to demand that authors clarify how they dealt with interactions in their study, including potential interactions that were not part of the final statistical model, and also whether a test was used to justify their exclusion. The main focus in this review was on whether interaction terms have been used and tested, and, if not, if this was discussed in the article. For the criterion to be fulfilled in this review it was sufficient that at least one interaction between two predictor variables was reported, but this does not establish whether authors have adequately described how they dealt with all potentially important interactions in their study. This review also documents how many articles presented stratified results, and any tests used to determine differences in effect between factor levels.

### Collinearity of independent variables

If two predictor variables are highly correlated they have the potential to bias estimates of the relationship between each predictor variable and the outcome variable. It is, therefore, important to consider this in any statistical analysis. To fulfill the criterion in this review, the authors must have specified that they used a test for collinearity, e.g. Spearman’s rho, and also provided the result of the test.

### Validation of the statistical model

To verify whether the statistical model is useful for estimates of the relationship between the outcome variable and the predictor variables, a validation of the relationship is required. Common techniques for internal validation are: i) split the dataset into two parts, where the first part is used to estimate model coefficients and the other part to calculate goodness of fit of the model [2, 5]; ii) redo the analysis on a different sample, or iii) use jackknife or bootstrap techniques. External validation, has rarely been used as a criterion in similar evaluations. This is difficult to demonstrate in public health research and was not therefore included here as a validation criterion.

### Statistical significance/confidence intervals

It is usually the case that a *p*-value is provided for estimates on the variable level but not always for the whole model. This review investigated whether measures of significance were provided on the variable level as well as for the statistical model as a whole. The importance of the significance for the model can be argued, but such information is always relevant to provide. This review also collected information about whether confidence intervals were provided for the effect of unemployment on health. It has been recommended many times that confidence intervals should be used instead of *p*-values in the presentation of the results [75], but confidence intervals are still not commonly used [12, 76]. It has also been recommended that authors present exact *p*-values and not only specify whether the *p*-value is below or above the significance level. Articles that provide non-exact *p*-values, i.e., those that only specify significance above or below a threshold, were identified. However confidence intervals provide the same information as exact *p*-values, and so articles that reported confidence intervals were assumed to have met this criterion.

### Coefficients in statistical models with relative differences

It is essential to present all coefficients in the statistical model, even if the main interest is limited to the effect of unemployment on health. To derive a valid estimate of this effect, other effect estimates might not correctly present their association with the outcome variable, nevertheless it is important to present these coefficients in order for proper judgement of the validity of the statistical analysis. This review evaluated the extent to which all coefficients are presented in statistical models with a relative measurement (odds ratio or prevalence ratio). Articles with absolute differences were not included because some of these statistical models are more complex, and all coefficients can therefore not be straightforwardly presented.

### Goodness of fit test

A statistical model will not fit the data perfectly. Goodness-of-fit measures can describe how well the model fits the observed values, and diagnostics such as residuals, leverage, and influential observations are also capable of providing such analysis [2].

### Selection of predictor variables/presentation of coefficients in the model

It is essential to provide some rationale for the inclusion of all variables in a statistical model. Among the most common reasons for including variables in a model are previous research which included the same or similar variables, and bivariate analyses of the candidate variables [2]. However it is common for articles to perform a multivariable analysis without presenting the mathematical model in full. Sometimes authors do not even mention which variables contribute significantly to the model. With regard to this issue, this review focused on the choice of predictor variables for the original model and the choice of predictor variables for the final model presented in the article. The selection of variables in the final model could be, for example, based on a decision to keep or remove non-significant variables. This review does not judge how well authors have succeeded to choose variables in preliminary and final models, but rather evaluates whether authors provided informed reasons for their choices. This is a similar but different criterion to that of the fitting procedure criterion.

### Coding of variables

Appropriate information about the variables in the statistical model is necessary in order to understand them. This includes detailed information about what the variable measures and the different outcomes of the variable [2]. Articles were graded as to whether they had either no faults, small faults, or major faults. Small faults means that there was a lack of detail provided although it was possible to determine how the variables are used and what the different values meant. In the articles which had major faults it was not possible to determine how all the variables were created and used.

### Fitting procedure

The selection of variables from the candidate variables can be performed in different ways. For linear regression analysis, forward inclusion, backward elimination, and best-subset are the most common methods for final fitting of the statistical model. How the selection was done needs to be explicitly stated in the article. It should also be made clear how the use of fitting procedures can change the significance levels and how adjustments were made in the article for this purpose.

### Methodological concerns discussed

To my knowledge, this criterion has not been discussed in previous methodological reviews. Most statistical models have weaknesses, and these can be due to the inability to measure all relevant confounders as well as a lack of validity in the assessment and measurement of confounding. It can also be because the statistical model simply does not fit the data. Because there are weaknesses with any analysis of individual data, it is important that this is addressed by the authors. A discussion about potential biases is expected, but it would also be valuable to discuss why the choice of statistical analysis method is best suited to the data. A discussion of the limitations of the statistical analyses is one of the 22 items on the checklist that the STROBE initiative presented in 2007 [24], and this has been brought up as an important issue by many researchers.

### Criteria quality score

To assess to what extent articles were fulfilling the criteria used in this evaluation, a score was created. For the twelve criteria listed above, each article was given a score of one if the criterion was fulfilled, with the exception of the criterion “statistical significance/confidence intervals”. For this criteria “statistical significance for statistical model”, “exact *p*-value presented” and “confidence interval presented for exposure”, each counted for a score of one. For the criterion “selection of variables”, to be met it was necessary to fulfill the criterion “selection of candidate variables discussed”. For “coding of variables”, only those articles with “major faults” failed to fulfil the criterion. For the criterion “interactions between independent variables”, it was necessary to fulfill “interaction terms used and coefficients tested”. The criteria quality score for an article was calculated as the proportion of fulfilled criteria among applicable criteria (with a maximum of 14 criteria for each article).

## Results

The criteria specified in the methods section were evaluated for the 41 articles in this review, and the results for each article are presented in Additional file 1, and summarized in Table 1. The characteristics of the articles have been presented in a previous review [68]. In that article, it was shown that over half (*n* = 24) of the studies were cross-sectional and the remaining articles had a longitudinal design (*n* = 17). Binary logistic regression (*n* = 21) was the most commonly used method for statistical analysis, and this was followed by methods based on other regression techniques (*n* = 18) such as fixed effects regression and multiple linear regression. Over half of the articles (*n* = 23) used a measure of relative difference to compare unemployed with employed individuals, while it was a little less common to use the absolute difference (*n* = 20) for the comparison. Two articles used both a relative and absolute difference measurement [34, 62]. All but one article that measured relative differences used odds ratios, and the single different manuscript used prevalence ratios [55].

One article used person-years for the statistical analysis, and this was therefore considered not applicable for the events-per-variable criterion [38]. Of the remaining 40 articles, only one failed to fulfill the events-per-variable criterion. This study presented results per country and the criterion was fulfilled for only one of the ten countries.

In 19 articles there were continuous variables in the statistical model, but none of these articles showed whether the continuous variables conformed to the linear gradient. Thus, none of the articles fulfilled the criterion for nonconformity with a linear gradient.

Interaction terms were included in the statistical model in 14 articles, and in all of these, the interaction terms were analyzed with a statistical test. In 27 articles, stratified results were presented. In three of these articles (11 %), tests for interaction were performed between factor levels. In seven articles (17 %), both interaction terms and stratified results were included. In seven articles (17 %), neither interaction terms nor stratified results were presented. In articles with stratified results but no interaction terms, a potential interaction between variables was rarely mentioned. From what was described in the articles, the stratification did not appear to be used in place of interaction terms.

Only one article (2.6 %) discussed and tested potential conflicts between independent variables due to collinearity. The article excluded two variables that were highly correlated with another independent variable, based on Spearman’s rho, from the extended statistical model [26]. In another article it was mentioned that two independent variables were collinear, but a test of collinearity was not provided in the article and the variables were also included in the analysis [41]. In two articles, only univariate analyses were performed and the criterion was therefore not applicable.

No statistical model was validated in any of the articles included in this review. However, three articles used sensitivity analysis or robustness checks of their statistical model, which are similar but not the same as validation [31, 32, 42]. Only two articles reported statistical significance for their models [40, 57]. In 14 articles (34 %), the *p*-value was only presented as being above or below the significance level. In three of the articles (14 %) that used logistic regression [36–38], this criterion was not fulfilled. In 17 articles (42 %), there were no confidence intervals presented for the effect of unemployment on health. Slightly less than half of the 23 articles (48 %) that used relative differences (odds or prevalence ratios) did not present all coefficients in the statistical model.

Nine articles (22 %) used a test to evaluate the fit of the statistical model. The coefficient of explanation (R^2^), which was used in six articles, was the most common test. Only two articles (9.5 %) that used logistic regression performed a goodness of fit test.

The selection of initial variables for the statistical model was discussed in 13 articles (32 %), while the final selection of variables was only discussed and justified in five articles (12 %).

Three articles (7 %) had major faults in the way in which they described the coding of their variables. There was, for example, a lack of description regarding education levels [53] and family composition [37]. In another 11 articles (27 %), the description of the coding of variables was also lacking, but to a less extent.

The fitting procedure was only described in three articles (7 %). In 31 articles (76 %), potential biases were discussed. A more thorough discussion which included limitations with the statistical model, was in six of these articles (15 %).

Out of all articles, eight (20 %) had a score for the criteria quality score of at least 0.5 and 23 (56 %) had a score of at least 0.4, with the highest score being 0.62.

## Discussion

The quality of the reporting and use of statistical analyses in articles that studied the effect of unemployment on health was poor. This is not unique given the focus of this review. The issue has also been addressed in previous analyses of methodological shortcomings in scientific articles. This review focused on twelve different criteria. The majority of the articles failed to fulfill most of the criteria, the main ones being conformity with a linear gradient (100 % of the articles), validation of the statistical model (100 %), collinearity of independent variables (97 %), fitting procedure (93 %), goodness of fit test (78 %), selection of variables (68 % for the candidate model; 88 % for the final model), and interactions between independent variables (66 %).

All but one article fulfilled the criterion for events per variable. However, in some of the articles in which the criterion were fulfilled, the authors were probably not aware of this, but it was achieved because of a large sample size and a restricted number of candidate independent variables. Previous reviews reported a potential problem with events per variable in the statistical model for 39–62 % of the included studies [2, 5, 11, 16, 17, 19], but exceptions to this are the reviews by Mantzavinis et al. that reported problems for only 10 % of the articles and by Kalil et al. that reported problems for 16 % of the articles [7, 14]. None of the articles with continuous variables provided evidence that their choice of a linear gradient for the continuous variables in the statistical model was valid. This criterion has been previously shown to be poorly met with at most 29 % of articles reporting such evidence [14]. Other evaluations report that only about 15 % of articles fulfill this criterion [2, 7, 9, 11, 16, 17, 19].

Few articles (34 %) included or discussed interaction terms, but those that did also tested the significance of the interactions. Similar results were presented in most previous reviews [2, 5, 7, 9, 11, 14, 16, 17, 19, 21], with one review showing that 45 % of the articles fulfilled the criterion [21] and one showing that fewer than 10 % fulfilled the criterion [11]. A majority of the articles reviewed here (66 %) presented stratified results, which to some extent can be considered to fill the role of checking for interactions in the statistical model, but this is not a sufficient reason for failing to discuss potential interactions in an article. Only two articles discussed collinear variables. Previous similar reviews also reported that few articles discuss the potential problems arising from collinear variables. It was only the review by Ottenbacher et al. (17 % of articles) that reported that this was fulfilled in over 10 % of the reviewed articles [2, 7, 11, 16, 17, 19, 21].

None of the articles reported that they had validated their statistical models, and this is consistent with previous similar reviews which showed that few articles reported that their models had been validated (at most 10 % in Tetrault et al.) [2, 7, 11, 16, 19, 21]. Only two articles provided explicit statistical significance for the model. In 14 articles, neither a confidence interval nor an explicit *p*-value was given; instead, a *p*-value was presented as simply below or above the significance level. Previous articles that have evaluated how frequently articles present confidence intervals in the logistic regression have reported similar proportions to the 14 % reported here. They include 26 % reported by Moss et al. and 29 % reported by Ottenbacher et al. [17, 19]. Despite being recommended by many authors to always report confidence intervals [75], it was not reported for most other methods.

About 48 % of the articles did not report all coefficients in the statistical models. In previous evaluations this criterion was fulfilled to a greater extent, even though these evaluations also required that confidence intervals were reported for the independent variables [7, 17]. Few articles (22 %) presented goodness of fit tests in this review, but this was more than in previous reviews [2, 7, 9, 11, 16, 19]. In three of these previous reviews, fewer than 5 % of the included articles reported such tests [2, 7, 16]. However, the higher rate of the use of goodness of fit tests in this review can mainly be explained by the inclusion of articles that do not use logistic regression.

Most of the articles may have had reasons for the inclusion of variables, but it is only possible to judge if this was the case if it is reported, and only 32 % of the articles in this review did so. The selection of variables has often been evaluated in previous reviews, and the number of articles that fulfill the criterion has varied from as high as 95 % [2] to as low as 15 % [21]. In general, previous reviews have presented a higher frequency of articles fulfilling the criterion than in this review [2, 5, 7, 9, 11, 14, 16, 17, 19, 21]. The importance of arguing for the final selection of variables has also been discussed previously, but no previous reviews have evaluated this. Only few (12 %) of the articles in this review commented on how the final selection of variables was performed. This is an area in which improved reporting is needed.

It should be straightforward to report how variables are coded, but only 34 % of the articles met this criterion. In four previous reviews, this coding was considered insufficient in 85 % or more of the included articles [2, 5, 7, 19], and at most, 84 % of the articles reported fulfilling the criterion [21] in previous reviews [2, 5, 7, 9, 11, 14, 16, 17, 19, 21]. The fitting of the model was only mentioned in 7 % of articles reviewed here. In previous similar reviews, this requirement was variously met in 27 to 65 % of the articles [2, 7, 17, 19].

Methodological concerns are highlighted in the STROBE statement [24]. To my knowledge, I am the first one to evaluate how well articles are fulfilling this criterion. I decided to include this because I recognized while reading the articles in this review that surprisingly little space was devoted to this important issue. The choice of method for analyzing the data is crucial for presenting reliable results. Only six (15 %) of the articles in this review discussed the choice of statistical model, and 24 % of the articles did not explicitly discuss limitations related to their analyses. No statistical model is “perfect”, and therefore it is important for authors to inform the reader about weaknesses even if they are difficult, or even impossible, to avoid.

Most of the criteria in this review were used previously, for example, by Bagley and colleagues [2] and Concato and colleagues [5]. Even if articles fail to fulfill some of the criteria in this review, their analyses might still be of high quality. However, failure to report on what has been done suggests that authors lack knowledge about the statistical methods used to analyze their data. This is critical because wrong statistical analyses could potentially lead to incorrect results and, consequently, to wrong conclusions. However this review did not attempt to evaluate whether the results from the statistical analyses were valid in the reviewed articles.

Only one article stated that individual study data were available online [31]. In general, the lack of information about how statistical analyses were performed made it impossible to review the validity of the study results. However, it was obvious in a few articles that the analysis of the data did not correspond to the research aims therefore bringing the validity of the results to question. There has been evidence presented for mistakes in the performance of statistical methods by, for example, Garcia-Berthou and Alcaraz who reported incongruences between test statistics and *p*-values [77]. Lucena et al. reported that 41 % of their 209 reviewed articles used inappropriate statistical methods [12].

There are other criteria related to the quality of the performed statistical analyses that would have been valuable to assess in this review. Among these, it would have been useful if the articles in this review had assessed the use of cut-offs for the variables in the model. The grouping of variables seemed to be well chosen in the articles, but nevertheless it is a major weakness that cut-offs were rarely explained or justified. The efficiency of analysis is improved if as much information as possible is given regarding the variables, making it important to provide the reasons for the chosen cut-off. Some of the criteria in this review are related to confounding effects. However, whether a variable is a confounder or an effect modifier cannot be interpreted only from the coefficients of, for example, a logistic regression. The general impression from the articles in this review is that some authors adopted a very superficial approach to the assessment of confounders. I am not aware of any studies that have evaluated articles based on this aspect, and I suggest that this would be a good topic for a future study.

The STROBE statement was developed as a guideline for the reporting of observational studies, and it has been recommended as a checklist for scientific journals [24]. Lancet is one of the journals that require that the STROBE checklist is submitted, but the other most reputed medical journal, the New England Journal of Medicine (NEJM), does not require it. Instead, NEJM demands that the criteria for statistical analysis listed by Bailar and Mosteller in 1988 are fulfilled [78]. However, neither the STROBE statement nor the criteria by Bailar and Mosteller require that the criteria brought up by, for example, Bagley and colleagues and Concato and colleagues are fulfilled and/or discussed in articles [2, 5]. Hence, current guidelines for publication are not sufficient to ensure that the criteria used in this review are fulfilled, and publication guidelines are in need of further improvement.

The aim of this article was not to propose a new guideline or to propose an extension of current guidelines. The criteria that I have evaluated could be considered for such guideline. The challenge of improving current guidelines, has to be taken up by a well-reputed group of experts, similar to the STROBE group (or even the STROBE group itself), as a consensus is very important for such guidelines to be well received. Some of the criteria I have listed I consider crucial that they have been well thought through, among them the criteria related to interactions, collinearity, and conformity with a linear gradient. Poor handling of such criteria may result in highly biased estimates. Other issues covering validation of the statistical model place a higher demand on the statistical competence of the authors but inadequacy in this area can bias the estimates and therefore this is an important issue. It is particularly surprising that the criteria for variable coding and selection are not addressed, as little statistical competence is required to do so. Such documentation should be integral to the implementation phase of the study.

If authors are overloaded with instructions, this may mean that some valuable studies are not published. Current guidelines, such as the STROBE statement [24], require that a checklist is filled in and submitted as supplementary file. My suggestion for an improvement is that such a checklist should be submitted regarding the statistical analyses. If a study has been performed to an acceptable level then documenting what has been done should not be difficult. This is not time demanding, and the value for other researchers is substantial as they will be able to check the analyses and conclusions. Although some articles may not be published if the guidelines are tightened as suggested here, this would improve the overall quality of published papers. However one reasonable argument against introducing further criteria such as suggested here, is that it may be difficult to stay within word limits. However on the other hand some information could be moved from main manuscript to a supplementary file to offset this. It might also be that some of the criteria would not demand additional analysis, but that it would be sufficient to simply report that the analysis was not done in the checklist, e.g. no internal validation was performed.

An additional value from a checklist is that if important issues are highlighted in the journals’ instructions to the authors, it is likely that the authors will be better prepared to deal with these issues and such instructions might even help the authors in their statistical analyses. It is also important in the publication process that the reviewers are capable of evaluating how well the authors have fulfilled the criteria. Thus, I recommend not only improving the guidelines for authors, but also asking reviewers to assess the extent to which articles fulfill the criteria for statistical analysis. It is important, of course, that such requirements are handled in such a manner as to not unduly burden the reviewers because the review process is already highly demanding of reviewers’ time.

## Conclusions

There are critical methodological shortcomings among the studies included in this review. It is difficult to obtain unbiased estimates for the relationship between the exposure and the outcome variable, but it is nevertheless clear that improvements from the current status of the quality of the documentation and performance of the statistical methods are needed.

My suggestion for increased quality is that journals demand that articles not only fulfill the criteria within the STROBE statement, but that they extend the requirements to include additional criteria so as to reduce the risk of drawing incorrect conclusions from research studies.

## Additional file

Additional file 1: **Methodological evaluation of the articles in this review.** (DOCX 26 kb)
