# Supplementary material for: Poor quality in the reporting and use of statistical methods in public health – the case of unemployment and health
Source: Arch Public Health. 2015 Nov 16;73:56. doi: 10.1186/s13690-015-0096-6 (PMC4645480; doi:10.1186/s13690-015-0096-6)
Supplement: Additional file 1: — Methodological evaluation of the articles in this review. (DOCX 26 kb) [file 13690_2015_96_MOESM1_ESM.docx]

**Additional file 1.** Methodological evaluation of the articles in this review

| **Country** | **Method** | **Meas** | **EPV** | **LG** | **INTER** | **COLL** | **VALID** | **SIG/CIs** | **COEFF** | **GFT** | **SELVAR** | **CODE** | **FIT** | **DISC** |
| --- | --- | --- | --- | --- | --- | --- | --- | --- | --- | --- | --- | --- | --- | --- |
| Australia [17] | LinReg  (non-trivial) | AD | Yes | NC | ITT, SRN | ND | SA | NSM, NP, CNP | n/a | R2 | SD, SN | SF | No | PB |
| Australia [26] | Path | AD | Yes | No | SRN | ND | No | NSM, CNP | n/a | CFI, TLI, RMSE | ND, SN | NF | Yes | PB |
| Australia [32] | LinReg  (non-trivial) | AD | Yes | No | SRN | ND | No | CNP | n/a | R2 | SD, SN | NF | No | No |
| Belgium [12] | Log | RD | Yes | NC, AG | IND | ND | No | NSM, NP, CNP | No | No | ND, SN | MF | No | No |
| Brazil [16] | Log | RD | Yes | NC, AG | ITT, SRN | ND | No | NSM, CIP | Yes | No | SD, SN | NF | No | PB |
| Canada [9] | LinReg & Log | AD, RD | Yes | NC, AG | SRN | ND | No | NSM, CIP | No | No | ND, SN | NF | No | PB |
| China [25] | Log  (non-trivial) | RD | Yes | NC, AG | SRN^a^ | ND | No | NSM, CIP | Yes | No | ND, SN | NF | No | PB |
| Croatia [38]^b^ | ANCOVA | AD | Yes | NC, AG | ITT | ND | No | NSM, NP, CNP | n/a | No | ND, SN | NF | No | PB |
| Europe [2] | Log | RD | No | NC | IND | ND | No | NSM, CIP | No | No | ND, SM | NF | SV | PB |
| Europe [6] | Log | RD | Yes | NC, AG | SRN | ND | SA | NSM, CIP | No | No | ND, SN | SF | No | PB |
| Europe [13] | Log  (non-trivial) | RD | n/a | NC, AG | SRN | ND | No | NSM, NP, CNP | No | No | SD, SN | NF | No | No |
| Europe [33] | Log | RD | Yes | NC, AG | ITT, SRT | ND | No | NSM, CIP | No | No | ND, SN | NF | No | PB |
| Europe [34] | Log | RD | Yes | NC, AG | ITT, SRN | ND | No | NSM, CIP | No | No | ND, SN | NF | No | PB |
| Europe [36] | Log | RD | Yes | No | SRN | ND | No | NSM, NP, CNP | Yes | R2 | SD, SN | NF | No | Yes |
| Finland [7] | DiD, Prop | AD | Yes | No | IND | ND | SA | NSM, NP, CNP | n/a | No | ND, SM | SF | No | No |
| Finland [41] | Log | RD | Yes | No | SRN | ND | No | NSM, CIP | No | No | ND, SN | NF | No | PB |
| Germany [23] | MCDR | RD | Yes | No | SRN | ND | No | NSM, CIP | No | No | ND, SN | SF | No | PB |
| Germany [35] | LinReg  (non-trivial | AD | Yes | No | SRN | ND | RC | NSM, NP, CNP | n/a | No | ND, SN | MF | No | PB |
| Great Britain [8] | LinReg  (non-trivial) | AD | Yes | No | ITT | ND | No | NSM, NP, CNP | n/a | No | SD, SN | NF | No | PB |
| Great Britain [10] | Log | RD | Yes | NC, AG | IND | ND | No | NSM, CIP | No | No | ND, SN | NF | No | No |
| Great Britain [14] | Reg | AD | Yes | No | IND | ND | No | NSM, CIP | n/a | No | SD, SN | NF | No | Yes |
| Great Britain [15] | IV, LinReg | AD | Yes | NC, AG | ITT | ND | No | NP, CNP | n/a | NE | ND, SN | SF | No | PB |
| Great Britain [28] | GLM | AD | Yes | No | SRN | ND | No | NSM, CIP | n/a | No | ND, SN | MF | No | No |
| Great Britain [29] | Log | RD | Yes | No | IND | ND | No | NSM, CIP | Yes | R2 | ND, SN | NF | No | PB |
| Great Britain [39] | Reg | AD | Yes | No | ITT | ND | No | NSM, CIP | n/a | No | ND, SN | NF | No | Yes |
| Norway [27] | Reg | AD | Yes | No | IND | ND | No | NSM, CNP | n/a | WT | ND, SN^c^ | SF | No | Yes |
| Poland [22] | Log | RD | Yes | NC, AG | SRN | ND | No | NSM, CIP | Yes | No | ND, SN | NF | No | PB |
| Slovakia [4] | Reg | AD | No | No^d^ | SRN | ND | No | NSM, NP, CNP | n/a | R2 | SD, SN | NF | No | No |
| Spain [3] | Log | RD | Yes | No | SRN | ND | No | NSM, CIP | Yes | No^e^ | ND, SN | NF | No | No |
| Spain [30] | Prev | RD | Yes | NC, AG | SRN | n/a | No | NSM, CIP | Yes | No | ND, SN | SF | No | PB |
| Sweden [1] | MNLog | RD | Yes | NC, AG | SRN | Yes | No | NSM, CIP | Yes | No | ND, SM | SF | No | No |
| Sweden [5] | Log | RD | Yes | NC, AG | ITT, SRT | ND | No | NSM, CIP | Yes | No | ND, SM | SF | NR | PB |
| Sweden [18] | Log | RD | Yes | NC | ITT, SRN | ND | No | NSM, CIP | No | No | ND, SN | NF | No | PB |
| Sweden [19] | ANOVA,  t-test^f^ | AD | Yes | NC | ITT | ND | No | NSM, NP, CNP | n/a | No | SD, SN | NF | No | PB |
| Sweden [20] | ANOVA,  t-test^f^ | AD | Yes | NC | ITT | ND | No | NSM, NP, CNP | n/a | No | SD, SN | NF | No | PB |
| Sweden [21] | Log | RD | Yes | No | SRN | n/a | No | NSM, CIP | Yes | No | SD, SN | NF | No | Yes |
| Sweden [24] | Log | RD | Yes | NC, AG | SRN | ND | No | NSM, CIP | Yes | No | ND, SN | SF | No | PB |
| Sweden [31] | Log | RD | Yes | No | SRN | ND | No | NSM, CIP | No | No | ND, SN | SF | No | PB |
| The Netherlands [37] | Log, LinReg | AD, RD | Yes | NC, AG | ITT, SRN | ND | No | NSM, CIP | Yes | No | SD, SM | NF | No | No |
| USA [11] | LinReg | AD | Yes | No | ITT | ND | No | NSM, NP, CNP | n/a | R2 | ND, SN | NF | No | PB |
| USA [40] | MNLog | RD | Yes | No | SRT | ND | No | NSM, CNP | Yes | No | SD, SN | NF | No | Yes |

**Note:** Number in parenthesis after country specification refers to listing in the reference list of this appendix.
**For all criteria, “**Yes” means that the criterion was fulfilled, “No” means that criterion was not fulfilled, and n/a means that the criterion was not applicable for the article.
**Country** refers to the country where study was performed (Europe refers to studies where at least two European countries participated); **Method** = the statistical method used for analyzing the relationship between health and unemployment (ANOVA = analysis of variance, DiD = difference in difference (similar to linear regression), GLM = generalized linear models, IV = instrumental variables, Log = logistic regression, LinReg = linear regression, MCDR = multivariate count data regression, MNLog = multinomial logistic regression, OL = ordinary logit, Prop = propensity scores, Reg = regression technique other than linear and logistic); **Meas** = effect measurement used for statistical analysis (AD = absolute difference, RD = relative difference); **EPV** = events per variable/overfitting; **LG** = conformity with a linear gradient (NC = no continuous variable in multivariate analysis, AG = age grouped); **INTER** = interactions between independent variables (IND = interactions not discussed in the article, ITT = interaction terms used and tested, SRN = stratified results presented but not tested, SRT = stratified results presented and tested); **COLL** = collinearity of independent variables (ND = not discussed); **VALID** = validation of the statistical model (RC = robustness check, SA = sensitivity analysis); **SIG/CIs** = presentation of significances and confidence intervals (CIP = confidence intervals presented for all independent variables in the main analysis, CNP = coefficients (and confidence intervals) not presented for all independent variables in the main analysis, NP = no explicit p-value presented, NSM = no significance for the model presented); **COEFF =** coefficients presented in statistical models with relative difference; **GFT** = results from goodness of fit test presented (CFI = comparative fit index, NE = not explained how goodness of fit test is performed in method section, R2 = coefficient of determination (pseudo, adjusted, or non-adjusted), RMSE = root mean square error of approximation, WT = Woodridge test, TLI = Tucker-Lewis index); **SELVAR** = selection of variables (SD = selection of candidate variables discussed, ND = selection of candidate variables not discussed, SM = final selection of variables motivated, SN = final selection of variables not motivated); **CODE** = coding of variables (NF = no faults, SF = small faults, MF = major faults); **FIT** = procedure for fitting the model to the final selection of variables (NR = variables not related to outcome or with a negligible effect on statistical model removed from model, SV = significant variables from univariate analyses (p < 0.10) and variables with significance in final model (p < 0.05) were selected without further explanation on how this was decided); **DISC** = methodological concerns discussed in the article (PB = potential bias discussed but not potential limitations with the statistical method).

^a^ It is mentioned in the method section that interaction terms were added to the model. However, there are none presented in the results. ^b^ ANCOVA was used. This is not an appropriate method for analyzing the outcome variable of the article because it is on an ordinal scale (5 levels). ^c^ BIC (assumed to be Bayesian Information Criterion) is presented in the tables but is not explained and interpreted in the article. ^c^ Linear regression requires a continuous independent variable. This study has only independent variables that are qualitative. ^d^ It is specified in the article that Hosmer-Lemeshow’s test is used, but no results are presented for this test. ^e^ It is unclear whether *t*-test or ANOVA has been used for the main analyses of the article. However, neither test is appropriate for analyzing the outcome variable of the article because it is on an ordinal scale (5 levels).

# References

1. Åhs A and Westerling R. Self-rated health in relation to employment status during periods of high and of low levels of unemployment. *Eur J Public Health*. 2006; 16: 294-304.

2. Alavinia SM and Burdorf A. Unemployment and retirement and ill-health: a cross-sectional analysis across European countries. *Int Arch Occup Environ Health*. 2008; 82: 39-45.

3. Artazcoz L, Benach J, Borrell C and Cortes I. Unemployment and mental health: Understanding the interactions among gender, family roles, and social class. *Am J Public Health*. 2004; 94: 82-8.

4. Bacikova-Sleskova M, van Dijk JP, Geckova AM, et al. The impact of unemployment on school leavers' perception of health. Mediating effect of financial situation and social contacts? *Int J Public Health*. 2007; 52: 180-7.

5. Backhans MC and Hemmingsson T. Unemployment and mental health-who is (not) affected? *Eur J Public Health*. 2012; 22: 429-33.

6. Bambra C and Eikemo TA. Welfare state regimes, unemployment and health: a comparative study of the relationship between unemployment and self-reported health in 23 European countries. *J Epidemiol Community Health*. 2009; 63: 92-8.

7. Böckerman P and Ilmakunnas P. Unemployment and Self-Assessed Health: Evidence from Panel Data. *Health Econ*. 2009; 18: 161-79.

8. Booker CL and Sacker A. Psychological well-being and reactions to multiple unemployment events: adaptation or sensitisation? *J Epidemiol Community Health*. 2012; 66: 832-8.

9. Breslin FC and Mustard C. Factors influencing the impact of unemployment on mental health among young and older adults in a longitudinal, population-based survey. *Scand J Work Env Hea*. 2003; 29: 5-14.

10. Brown J, Demou E, Tristram MA, Gilmour H, Sanati KA and Macdonald EB. Employment status and health: understanding the health of the economically inactive population in Scotland. *BMC public health*. 2012; 12.

11. Burgard SA, Brand JE and House JS. Toward a better estimation of the effect of job loss on health. *J Health Soc Behav*. 2007; 48: 369-84.

12. Burnay N, Kiss P and Malchaire J. Sociability, life satisfaction, and mental health according to age and (un)employment status. *Assessment and Promotion of Work Ability, Health and Well-being of Ageing Workers*. 2005; 1280: 347-52.

13. Cooper D, McCausland WD and Theodossiou I. Unemployed, uneducated and sick: the effects of socio-economic status on health duration in the European Union. *J R Stat Soc a Stat*. 2008; 171: 939-52.

14. Flint E, Bartley M, Shelton N and Sacker A. Do labour market status transitions predict changes in psychological well-being? *Journal of epidemiology and community health*. 2013; 67: 796-802.

15. Gathergood J. An instrumental variable approach to unemployment, psychological health and social norm effects. *Health Econ*. 2013; 22: 643-54.

16. Giatti L, Barreto SM and Cesar CC. Unemployment and self-rated health: Neighborhood influence. *Soc Sci Med*. 2010; 71: 815-23.

17. Green F. Unpacking the misery multiplier: How employability modifies the impacts of unemployment and job insecurity on life satisfaction and mental health. *J Health Econ*. 2011; 30: 265-76.

18. Hammarström A, Gustafsson PE, Strandh M, Virtanen P and Janlert U. It's no surprise! Men are not hit more than women by the health consequences of unemployment in the Northern Swedish Cohort. *Scandinavian journal of public health*. 2011; 39: 187-93.

19. Hultman B and Hemlin S. Self-rated quality of life among the young unemployed and the young in work in northern Sweden. *Work*. 2008; 30: 461-72.

20. Hultman B, Hemlin S and Hörnquist JO. Quality of life among unemployed and employed people in northern Sweden. Are there any differences? *Work*. 2006; 26: 47-56.

21. Janlert U and Hammarström A. Which theory is best? Explanatory models of the relationship between unemployment and health. *BMC public health*. 2009; 9: 235.

22. Kaleta D, Makowiec-Dabrowska T and Jegier A. Employment Status and Self Rated Health. *Int J Occup Med Environ Health*. 2008; 21: 227-36.

23. Kroll LE and Lampert T. Unemployment, Social Support and Health Problems Results of the GEDA Study in Germany, 2009. *Dtsch Arztebl Int*. 2011; 108: 47-U14.

24. Lindström M. Psychosocial work conditions, unemployment and self-reported psychological health: a population-based study. *Occup Med-Oxford*. 2005; 55: 568-71.

25. Luo J, Qu Z, Rockett I and Zhang X. Employment status and self-rated health in north-western China. *Public health*. 2010; 124: 174-9.

26. Olesen SC, Butterworth P, Leach LS, Kelaher M and Pirkis J. Mental health affects future employment as job loss affects mental health: findings from a longitudinal population study. *BMC Psychiatry*. 2013; 13.

27. Østhus S. Health effects of downsizing survival and job loss in Norway. *Soc Sci Med*. 2012; 75: 946-53.

28. Popham F and Bambra C. Evidence from the 2001 English Census on the contribution of employment status to the social gradient in self-rated health. *J Epidemiol Community Health*. 2010; 64: 277-80.

29. Popham F, Gray L and Bambra C. Employment status and the prevalence of poor self-rated health. Findings from UK individual-level repeated cross-sectional data from 1978 to 2004. *Bmj Open*. 2012; 2.

30. Puig-Barrachina V, Malmusi D, Martinez JM and Benach J. Monitoring Social Determinants of Health Inequalities: The Impact of Unemployment among Vulnerable Groups. *Int J Health Serv*. 2011; 41: 459-82.

31. Reine I, Novo M and Hammarström A. Unemployment and ill health - A gender analysis: Results from a 14-year follow-up of the Northern Swedish Cohort. *Public health*. 2013; 127: 214-22.

32. Richardson S, Lester L and Zhang GY. Are Casual and Contract Terms of Employment Hazardous for Mental Health in Australia? *J Ind Relat*. 2012; 54: 557-78.

33. Roos E, Burström B, Saastamoinen P and Lahelma E. A comparative study of the patterning of women's health by family status and employment status in Finland and Sweden. *Soc Sci Med*. 2005; 60: 2443-51.

34. Roos E, Lahelma E, Saastamoinen P and Elstad JI. The association of employment status and family status with health among women and men in four Nordic countries. *Scand J Public Health*. 2005; 33: 250-60.

35. Schmitz H. Why are the unemployed in worse health? The causal effect of unemployment on health. *Labour Econ*. 2011; 18: 71-8.

36. Schröder M. Jobless now, sick later? Investigating the long-term consequences of involuntary job loss on health. *Adv Life Course Res*. 2013; 18: 5-15.

37. Schuring M, Burdorf A, Kunst A, Voorham T and Mackenbach J. Ethnic differences in unemployment and ill health. *Int Arch Occup Environ Health*. 2009; 82: 1023-30.

38. Sersic DM, Sverko B and Galesic M. Unemployment and dimensions of subjective health: Exploring moderating effects of age. *Stud Psychol*. 2005; 47: 221-34.

39. Steele F, French R and Bartley M. Adjusting for Selection Bias in Longitudinal Analyses Using Simultaneous Equations Modeling The Relationship Between Employment Transitions and Mental Health. *Epidemiology*. 2013; 24: 703-11.

40. Strully KW. Job Loss and Health in the Us Labor Market. *Demography*. 2009; 46: 221-46.

41. Virtanen P, Liukkonen V, Vahtera J, Kivimäki M and Koskenvuo M. Health inequalities in the workforce: the labour market core-periphery structure. *International journal of epidemiology*. 2003; 32: 1015-21.
